# Tracking intra‐ and inter‐organelle signaling of mitochondria

**DOI:** 10.1111/febs.15103

**Published:** 2019-11-11

**Authors:** Corina T. Madreiter‐Sokolowski, Jeta Ramadani‐Muja, Gabriela Ziomek, Sandra Burgstaller, Helmut Bischof, Zhanat Koshenov, Benjamin Gottschalk, Roland Malli, Wolfgang F. Graier

**Affiliations:** ^1^ Gottfried Schatz Research Center, Molecular Biology and Biochemistry Medical University of Graz Austria; ^2^ Department of Health Sciences and Technology ETH Zurich Schwerzenbach Switzerland; ^3^ BioTechMed Graz Austria

**Keywords:** calcium, intracellular signaling, mitochondria, mitochondria‐associated ER membranes, mitochondrial membrane potential, mitochondrial structure, potassium

## Abstract

Mitochondria are as highly specialized organelles and masters of the cellular energy metabolism in a constant and dynamic interplay with their cellular environment, providing adenosine triphosphate, buffering Ca^2+^ and fundamentally contributing to various signaling pathways. Hence, such broad field of action within eukaryotic cells requires a high level of structural and functional adaptation. Therefore, mitochondria are constantly moving and undergoing fusion and fission processes, changing their shape and their interaction with other organelles. Moreover, mitochondrial activity gets fine‐tuned by intra‐ and interorganelle H^+^, K^+^, Na^+^, and Ca^2+^ signaling. In this review, we provide an up‐to‐date overview on mitochondrial strategies to adapt and respond to, as well as affect, their cellular environment. We also present cutting‐edge technologies used to track and investigate subcellular signaling, essential to the understanding of various physiological and pathophysiological processes.

## Graphical abstract

This review provides an up‐to‐date overview of the intra‐ and interorganelle signaling within mitochondria, highlighting their role as highly specialized working units and signaling hubs. Moreover, this review presents cutting‐edge technologies to track the kinetics and dynamics of these molecular processes, which are essential to the understanding of various physiological and pathophysiological processes. For instance, super‐resolution microscopy enables us to follow morphological changes within mitochondria under assorted conditions.

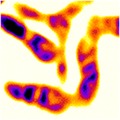

**Abbreviations**
CJ: cristae junction
CM: cristae membrane
DRP1: dynamin‐related protein 1
dSTORM: direct stochastic optical reconstruction microscopy
EMRE: essential MCU regulator
ETC: electron transport chain
FADH_2_: flavin adenine dinucleotide
FRET: Förster resonance energy transfer
GE: genetically encoded
GPS2: G protein pathway suppressor 2
IMM: inner mitochondrial membrane
IP3R: inositol triphosphate receptor
LETM1: leucine zipper‐EF‐hand‐containing transmembrane protein 1
LIT: Light‐inducible tethering
MAMs: mitochondria‐associated ER membranes
MCU: mitochondrial calcium uniporter
MFN1: mitofusin 1
MFN2: mitofusin 2
MICOS complex: mitochondrial contact site and cristae organizing complex
MICU1: mitochondrial calcium uptake 1
MICU2: mitochondrial calcium uptake 2
mPTP: mitochondrial permeability transition pore
mtUPR: mitochondrial unfolded protein response
NADH: nicotinamide adenine dinucleotide
OMA1: metalloprotease‐related protein 1
OMM: outer mitochondrial membrane
OPA1: optic atrophy 1
OXPHOS: oxidative phosphorylation
PALM: photo‐activated localization microscopy
PE: phosphatidylethanolamine
PS: phosphatidylserine
ROS: reactive oxygen species
SIM: structured illumination microscopy
STED: stimulated emission depletion
TMRM: tetramethylrhodamine methyl ester
TOM: translocase of the outer membrane
UCP2: uncoupling protein 2
UCP3: uncoupling protein 3
VDAC: voltage‐dependent anion channel
YME1L: ATP‐dependent metalloprotease
Δψ_m_: Mitochondrial membrane potential

## Introduction

**Figure 1:**
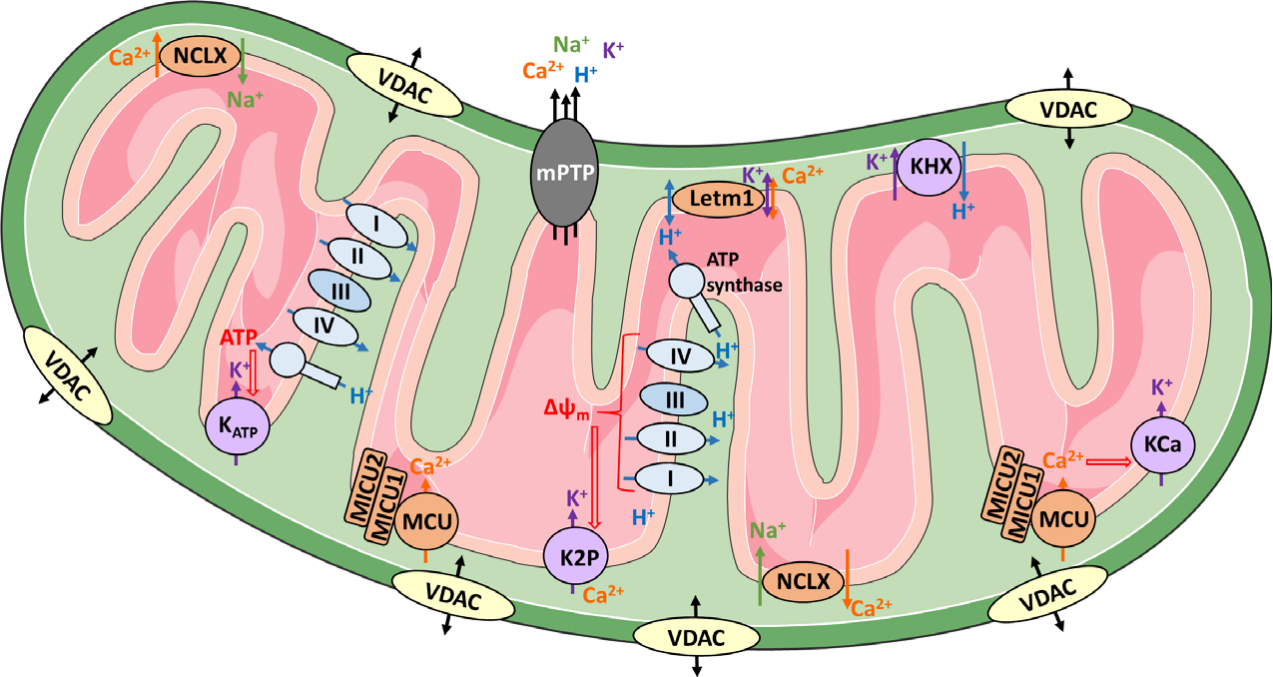
An overview of various types of mitochondrial ion channels. The OMM is largely permeable due to the VDAC, which allows the transport of small metabolites and ions across the OMM. In contrast, ion transport across the IMM has to be highly constricted. Proton pumping from the mitochondrial matrix into the intermembrane space generates the Δψ_m_, boosting ATP generation and regulating the transfer of ions across the IMM. K^+^ influx is modulated by the Δψ_m_ triggering K^+^ influx via two‐pore domain K^+^ (K2P) channels and by mitochondrial ATP production affecting ATP‐sensitive K^+^ channels (K_ATP_). Moreover, mitochondrial Ca^2+^ levels boost mitochondrial K^+^ uptake by affecting the activity of Ca^2+^‐activated K^+^ (KCa) channels. Extrusion of K^+^ is ensured by the K^+^/H^+^ transporter (KHX). The homeostasis of mitochondrial Ca^2+^ levels is also highly regulated. The MCU ensures MICU and gets tightly controlled by various proteins, including the gatekeepers MICU1 and MICU2. Furthermore, mitochondrial Ca^2+^ level is kept in check through an exchange with other ions by, for instance, the NCLX. Moreover, the LETM1 was proposed to act as Ca^2+^/H^+^ and/or K^+^/H^+^ antiporter, in either cases influencing Ca^2+^ influx and extrusion. Tight control of Ca^2+^ homeostasis is essential, since overwhelming accumulation of Ca^2+^ induces death‐bringing opening of the mPTP.

Mitochondria have undergone a drastic transition from free‐living bacteria to fundamental compartments within eukaryotic cells 1. Therefore, the mitochondria's broad field of contributions, ranging from production of ATP by oxidative phosphorylation (OXPHOS), buffering of Ca^2+^, and contribution to various cellular signaling pathways, was supplemented with highly dynamic structural and functional adaptations 2, 3. Stretching throughout the cell as a highly dynamic network along the cytoskeleton, mitochondria are constantly undergoing fusion and fission to meet the requirements of cellular subdomains under various conditions 2, 3, as discussed in our ‘Mitochondria as highly specialized working unit’ section. Besides by structure and shape, mitochondria's activity is largely controlled by the homeostasis of ions (Fig. 1), including H^+^ 4, 5, 6, K^+^ 7 and Ca^2+^ 8. These ions are not just powerful tools to fine‐tune mitochondrial activity, but also operate as messengers for intra‐organelle and intercellular communication, as explained in the chapter ‘Fine‐tuning of mitochondrial activity’. While fulfilling their multiple tasks, mitochondria strongly rely on the support of their cellular environment. This results in a busy interplay between mitochondria and various organelles 9, as summarized in ‘Mitochondria as signaling hubs: Give&Get’. Having the majority of proteins encoded by nuclear DNA makes a constant communication between mitochondria and the nucleus indispensable 10, for instance. Moreover, mitochondria form highly specialized signaling hubs with the endoplasmic reticulum (ER) to ensure and control lipid and Ca^2+^ transfer in restricted subdomains 11. Therefore, it is obvious that interplay between mitochondria and different cellular compartments takes place at specific contact sites or through the exchange of second messengers. To track these subcellular processes, cutting‐edge techniques are required to make investigation possible, including high‐resolution microscopy as well as highly sensitive organelle‐targeted biosensors. We provide an overview about techniques that enable us to study all of these processes at each chapter as well as in the table (Table 1), highlighting the importance of technological progress to reveal further mysteries about our cellular powerplants.

**Table 1:** Technical approaches to track mitochondrial structure, activity, and interorganelle interplay.

Technical approach	Advantages/disadvantages	References
Mitochondrial structure and shape		
Fluorescence microscopy		
Confocal microscopy	○ Conventional resolution of ~ 280 nm + Imaging of living cells and fixed cells possible − Resolution of maximal 150 nm (4Pi)	230
SIM	+ Live cell and time lapse imaging + Superior spatial resolution over confocal microscopy + Analysis of submitochondrial structures	66
STED		
PALM/dSTORM	+ Highest spatial resolution possible with fluorescence microscopy + Easy specific targeting with high labeling density using fluorescent marker − Fixation of the sample is often necessary − Temporal resolution of PALM displays a problem for moving structures in living cells	65
Electron microscopy	+ Precise analysis of submitochondrial structures possible + Very high spatial resolution + Immunogold preparation allows protein localization but lacks in labeling density − Fixation and embedding of the sample necessary	20
Mitochondrial energy production		
Oroboros O2k	○ Oxygen consumption is measured by a polarographic oxygen electrode + Analysis of cells, tissues, and isolated mitochondria possible + Sequential injection/titration of compounds possible − Not suitable for high‐throughput screening	74
Seahorse technology	+Oxygen consumption and extracellular acidification are measured in parallel by fluorescent sensors + Analysis of adherent cells, suspensions cells, permeabilized cells, isolated mitochondria, and—using specific tissue plates—tissues possible − Only four injections possible − 24‐ and 96‐well‐based assay platform	75
GE ATP probes	+ Organelle‐targeting makes analysis of ATP levels in various cellular compartments possible in real time − Proper transfection/infection efficiency is required	76, 77
ATeams		
pH_mito_		
GE pH probes		
mtAlpHi	○ Organelle‐targeted pH sensor that allows pH measurements in the lumen of mitochondria + Excitation of the probe at 498 nm reduces phototoxicity − Sensor provides intensiometric read‐out, hampering pH quantification − Proper transfection/infection efficiency is required	99
SypHer	○ Organelle‐targeted pH sensor that allows pH measurements in the lumen of mitochondria + Excitation at 430 nm and 480 nm with the detection of a constant emission wavelength at 530 nm allows a ratiometric read‐out and an easy pH quantification − Excitation of the probe at 430 nm might cause phototoxicity − Proper transfection/infection efficiency is required	92
Fluorescent dyes		
BCECF	○ Chemical pH sensor allowing global intracellular pH measurements + Cell loading with BCECF‐AM yields high fluorescent cell number + pKa of ~ 6.98 is close to the cytosolic pH + Excitation at 440 and 490 nm with the detection of a constant emission wavelength at 530 nm allows a ratiometric read‐out and an easy pH quantification − Excitation of the probe at 430 nm might cause phototoxicity − Presence of cellular esterases required for BCECF‐AM to BCECF cleavage	101
SNARF	○ Chemical pH sensor allowing global intracellular pH measurements + Cell loading with SNARF‐AM yields high fluorescent cell number + pKa of ~ 7.5 is close to the cytosolic intracellular pH + Excitation at ~ 500 nm with the detection of two emission wavelengths at 580 and 640 nm allows a ratiometric read‐out and an easy pH quantification + Long excitation wavelength of the probe reduces phototoxicity − Presence of cellular esterases required for SNARF‐1‐AM to SANRF‐1 cleavage − Although global intracellular staining, probe was used to measure pH_mito_ using high‐resolution microscopy − Measurements require a sophisticated microscope setup due to separation of two emission wavelengths	102
Mito‐pH	○ Chemical pH sensor allowing specific pH_mito_ measurements + Cell loading with Mito‐pH yields high fluorescent cell number + pKa of ~ 7.33 is close to the cytosolic pH + Excitation at 490 and 560 nm with the detection of emission wavelengths at 520 and 600 nm allows a ratiometric read‐out and an easy pH quantification − Long excitation wavelengths of the probe reduce phototoxicity	103
Δψm		
Fluorescent dyes		
TMRM	○ Monochromatic dye (λ_ex_ = 555 nm, λ_em_ = 570 nm) for semiquantitative analysis of Δψ_m_ + Cell loading with TMRM yields high fluorescent cell number − Mitochondria with low Δψm are possibly not stained and cannot be measured − Alteration of cellular respiration and binding to mitochondrial membrane might affect results	107
TMRE	○ Monochromatic dye (λ_ex_ = 549 nm, λ_em_ = 574 nm) for semiquantitative analysis of Δψ_m_ + Cell loading with TMRE yields high fluorescent cell number − Mitochondria with low Δψ_m_ are possibly not stained and cannot be measured − Alteration of cellular respiration and binding to mitochondrial membrane might affect results	105
JC‐1	○ Chemical, ratiometric dye (λ_ex_ = 488 nm, λ_em_ = 530 and 595 nm) for semiquantitative analysis of Δψ_m_ + All mitochondria are stained, independent of their Δψ_m_ + Cell loading with JC‐1 yields high fluorescent cell number − Photosensitive − Fluorescence may be changed independently of Δψ_m_ by, for instance, H_2_O_2_ or disturbed equilibrium between monomers and aggregates	106
Mitochondrial K^+^ homeostasis		
Patch‐clamp	+ Gold standard method for K^+^ fluctuation measurements + Very sensitive method − Isolation of mitochondria and preparation of mitoblasts required to measure K^+^ fluctuations across the IMM − Usage of isolated mitochondria might be far from the physiologic intracellular situation	118, 126, 128, 137
GE K^+^ probes		
GEPII	○ Organelle‐targeted K^+^ sensor that allows K^+^ measurements in the lumen of mitochondria + EC_50_ of 60.95 mm is suitable for measurements of [K^+^] within the mitochondrial matrix + Excitation at 430 nm with the detection of two emission wavelengths at 475 and 525 nm allows a ratiometric read‐out and an easy K^+^ quantification − Excitation of the probe at 430 nm might cause phototoxicity − Proper transfection/infection efficiency is required − Measurements require a rather sophisticated microscope setup as two emission wavelengths have to be properly separated	111
Mitochondrial Ca^2+^ homeostasis		
Fluorescent dyes		
Fura‐2	○ Indirect measurement of mitochondrial Ca^2+^ movement + Simple experimental preparation − Experimental preparation limited to simple cellular incubation − Not suitable for direct measurement of intra‐organelle Ca^2+^	174
Fluo‐3/Fluo‐4	○ Indirect measurement of mitochondrial Ca^2+^ movement + Simple experimental preparation − Significant leak in certain cell types resulting in lowered Ca^2+^ signals	179
Rhod‐2	+ AM ester dye that allows for mitochondria‐specific Ca^2+^ measurement − Only suited to short experimental protocols	180
GE Ca^2+^ probes		
4mtD3cpv	○ Organelle‐targeted, FRET‐based (λ_ex_ = 430 nm, λ_em_ = 480 and 535 nm) mitochondrial Ca^2+^ sensor ○ *K*_D_ for Ca^2+^ around 600 nm + Highly sensitive cameleon with a wide monitoring range − Proper transfection/infection efficiency is required	185
4mtD1GO‐CAM	○ Organelle‐targeted, FRET‐based (λ_ex_ = 477 nm, λ_em_ = 510 and 560 nm) mitochondrial Ca^2+^ sensor ○ *K*_D_ for Ca^2+^ around 1.53 μm + Red‐shifted cameleon with a wide monitoring range + Very well suitable for combination with other organelle‐targeted Ca^2+^ indicators and to be used simultaneously with Fura‐2 to correlate cytosolic and mitochondrial Ca^2+^ signals − Proper transfection/infection efficiency is required	186
mtGEM ‐GECO1	○ Organelle‐targeted, FRET‐based (λ_ex_ = 394 nm, λ_em_ = 455 and 511 nm) mitochondrial Ca^2+^ sensor ○ *K*_D_ for calcium around 340 nm + Very well suitable for combination with other organelle‐targeted Ca^2+^ indicators − Proper transfection/infection efficiency is required	187
mtCAR‐GECO1	○ Organelle‐targeted, intensiometric‐based (λ_ex_ = 565 nm, λ_em_ = 620 nm) mitochondrial Ca^2+^ sensor + Very well suitable for combination with other organelle‐targeted Ca^2+^ indicators − Proper transfection/infection efficiency is required	188

## Mitochondria as highly specialized working units

### Dynamic changes in structure and shape of mitochondria

The mitochondrial network is a highly specialized working unit capable of undergoing dynamic adaptation in order to meet metabolic needs and to allow internal and external signaling 2, 3. Thereby, constant fission and fusion of the inner (IMM) and outer mitochondrial membrane (OMM) play a crucial role in maintaining mitochondrial integrity. The fusion and fission homeostasis affects the opening probability of the mPTP, facilitating uncontrolled efflux of ions 12, as well as oxidative capacity 13, 14, production of reactive oxygen species (ROS) 15, mitophagy 16, apoptosis 17, 18, Ca^2+^ signaling 18, 19, and mitochondrial interactions with other cell compartments 20, 21, 22.

Mitochondrial fission is mainly mediated by the cytosolic dynamin‐related protein 1 (DRP1). It binds to the OMM and constricts the mitochondrion, an action facilitated by its GTPase activity 23. Furthermore, dynamin is necessary to achieve complete fission, as DRP1 activity leads to constriction, but not cleavage, during the fission process 24, 25. Nevertheless, it is still unknown whether a specific process or piece of protein machinery is necessary for the cleavage of the IMM.

Mitochondrial fusion is mainly driven by the proteins optic atrophy 1 (OPA1) and dynamin‐like protein mitofusin 1 and 2 (MFN1 and MFN2) 26, 27. MFN1 and MFN2 are located at the OMM and serve to facilitate fusion of two organelles. The necessary energy to fuse membranes is provided predominantly by the GTPase activity of MFN1 28. Extracellular‐signal‐regulated kinase is able to reduce MFN1 activity by phosphorylation, resulting in the fragmentation of mitochondria 29. Analogous to the OMM‐located MFN1 and MFN2, OPA1 is one of the main regulators responsible for the fusion of the IMM 30. Two forms of OPA1, L‐OPA1 and S‐OPA, are kept in balance by the metalloprotease‐related protein 1 (OMA1) and ATP‐dependent metalloprotease YME1L via proteolytic cleavage of the IMM bound L‐OPA1 to soluble S‐OPA1. A predominance of the L‐form yields mitochondrial fusion, while metabolic stimuli or cell stress signals activate L‐OPA1 proteolysis by OMA1 and YME1L, respectively, resulting in fission 31, 32.

Recently, intermitochondrial signaling by either nanotubes or direct interaction via intermitochondrial junctions has been intensively studied. These intermitochondrial contact sites have a high electron density and are coordinated pairs of cristae oriented orthogonally to the OMM in two adjoined mitochondria 33. Nano tunnels are either a result of stalled and incomplete fission events 34 or are *de novo* generated mitochondrial protrusions formed by members of the kinesin family, like the protein kinesin‐1 heavy chain along the cellular tubulin cytoskeleton 35. These intermitochondrial contact sites are thought to transmit Ca^2+^ or apoptotic signals across mitochondria 36 or even a coupling of the mitochondrial membrane potential (Δψ_m_) of neighboring mitochondria 37. Thereby, kissing and nanotunneling of mitochondria represent an alternative form of intermitochondrial communication 38, 39. Notably, exchange of, for instance, matrix proteins through nanotunnels follows a slower kinetic compared to conventional fusion most likely due to their small diameter of < 100 nm 38.

The IMM is morphologically separated into two compartments, divided by the cristae junction (CJ): (a) the inner boundary membrane directly facing the inner leaflet of the OMM; and (b) the cristae membrane (CM), forming the protrusions and invaginations of the IMM into the mitochondrial matrix 40, 41. Both compartments differ in protein composition and functional activity 42, 43. OPA1 is involved in the stabilization of the CJ and is interconnected with the mitochondrial contact site and cristae organizing complex (MICOS complex) 44. Loss of OPA1 by knockdown or knockout leads to widened cristae lumen and CJ 17, 45, reduction of IMM potential 46, increase in basal mitochondrial Ca^2+^ levels 47, and apoptosis induction 48. Similar to OPA1, the inner boundary membrane localized mitochondrial Ca^2+^ uptake 1 (MICU1) is involved in CJ stabilization, maintenance of IMM potential, and cytochrome c restriction to the cristae lumen. The Ca^2+^ sensing ability of MICU1 and its thereby affected quaternary structure might influence the CJ stability and permeability 49. The MICOS complex is composed of several subunits with mitofilin representing the biggest 50. MICOS proteins are especially enriched in the CJ and form IMM‐IMM and IMM‐OMM contact sites with proteins like OPA1 44, 51, translocase of the outer membrane (TOM) 52, 53 and S‐adenosylmethionine synthetase 54 to ensure CJ biogenesis and stabilization. The structure of the CJ also restricts the F_O_F_1_‐ATP synthase and the respiratory chain complexes to the CM, leading to a closed compartment of the cristae lumen 55, 56. Since the OMM is generally permeable due to voltage‐dependent anion channel (VDAC) 57, 58, the CJ seems to form a diffusion barrier for protons, creating an isolated space for the activity of F_O_F_1_‐ATP synthase and respiratory chain complexes with lateral pH gradients 59. Computational models and *in vitro* experiments have shown that low pH causes cristae invaginations of the IMM based on the electrostatic induced negative curvature of the outer CM leaflet 60, 61. Besides the direct involvement of the F_O_F_1_‐ATP synthase in cristae formation and morphology 42, 56, 62, an indirect effect of CM localized respiratory supercomplexes mediated by the decreased pH in the CJ might be an important factor in cristae biogenesis and shape definition 63.

#### Techniques to analyze structure and shape

While the current knowledge about the ultrastructure of mitochondria relies mainly on electron microscopy data, recent advancements in fluorescence microscopy, in particular super‐resolution microscopy techniques like structured illumination microscopy (SIM), stimulated emission depletion (STED), or photo‐activated localization microscopy (PALM), enable researchers to visualize and investigate mitochondrial ultrastructure in living cells.

direct stochastic optical reconstruction microscopy (dSTORM) is an alternative to immunogold labeling and electron microscopy for analysis of submitochondrial localization of proteins like the F_O_F_1_‐ATP synthase 64 or uncoupling protein 4 (UCP4) with high spatial resolution 65.

However, dSTORM is not suitable for live cell imaging and PALM approaches in live cells do not reach the necessary temporal resolution necessary to analyze dynamics of the IMM. Therefore, STED 66 or SIM 67 microscopy have been used to image, for instance, binding of fluorescent dyes dependent on Δψ_m_, such as Mitotracker Green^FM^ and tetramethylrhodamine methyl ester (TMRM), to the IMM. By using these techniques, cristae structure can be resolved and dynamic rearrangement of the IMM can be investigated due to high temporal resolution 67, 68. The dynamics of the IMM in regions of close mitochondria–ER contact could be analyzed, for instance. While silencing of OPA1 reduced IMM kinetics globally, ER Ca^2+^ release decreased IMM kinetics exclusively in mitochondrial areas in close proximity to the ER, pointing to a Ca^2+^‐regulated mechanism of IMM rearrangement 67. The submitochondrial dynamic and MICU1‐dependent relocalization of mitochondrial Ca^2+^ uniporter (MCU) and UCP2 upon ER‐Ca^2+^ release form the entire IMM into the inner boundary membrane, which was visualized recently using SIM 49. Similar observations of MCU inner boundary membrane localization under oxidizing conditions were made with STED and SIM showing the superior performance of super‐resolution over conventional microscopy in live cells 69.

The STED technique has been used to visualize spiral MICOS‐cluster arrays along the inner boundary membrane of yeast and human mitochondria, making an estimation of mitochondrial microarchitecture in combination with the use of immunogold labeling possible 43, 70. However, while STED microscopy achieves better resolution than SIM, it comes with the drawback of generally higher excitation intensity, leading to photobleaching and toxicity and, thus, limited acquisition time. SIM can be used with low excitation intensities, high frame rate, and over extended periods of time 71. Also, nonsaturation fluorescence microscopy along with intensive deconvolution was used to analyze the cristae kinetics in live cells and to distinguish between the IMM and matrix structures 68.

### Energy production as principal task

The mitochondria's core working unit is the mitochondrial respiratory chain located in the IMM within the cristae 55, 56. Oxidation of glycolysis derived pyruvate and nicotinamide adenine dinucleotide (NADH) by mitochondria yields about 15 times more ATP than glycolysis itself 72. Thereby, acetyl coenzyme A, derived from oxidative decarboxylation of pyruvate or beta‐oxidation, fuels the citric acid cycle to produce NADH and flavin adenine dinucleotide (FADH_2_).

These reducing equivalents serve as electron donors for the electron transport chain (ETC), consisting of NADH dehydrogenase (complex I), succinate dehydrogenase (complex II), ubiquinone, cytochrome bc1 complex (complex III), and cytochrome c and cytochrome c oxidase (complex IV). After transfer of electrons, derived from the NADH and FADH_2_ as hydrogen molecules, to the ETC via complex I and II, the electron transport through complex I to complex IV is coupled to proton pumping from the mitochondrial matrix into the locally restricted cristae lumen. This process causes a negative charge in the matrix and a positive charge in the IMS resulting in an electrochemical gradient, used for the proton transport back from the IMS into the mitochondrial matrix through ATP synthase (complex V). The released energy is, finally, utilized by F_O_F_1_‐ATP synthase to gain the cellular energy carrier, ATP, by phosphorylation of adenosine diphosphate 73.

#### Techniques to measure activity of mitochondrial activity

Besides a broad range of biochemical approaches such as western blot, ROS, and ATP assays, there are specific measurements for mitochondrial bioenergetics available. Based on a polarographic oxygen electrode measuring the concentration and consumption of oxygen before and after injection of various substrates, the Oroboros O2k has been in use since the 1990s. This machine offers the possibility to analyze cells, tissues as well as isolated mitochondria with high resolution. Furthermore, sequential injection and titration of compounds inhibiting different complexes of the mitochondrial respiratory chain or boosting maximal mitochondrial activity can be done during the ongoing measurement. However, the Oroboros O2k is not suitable for high‐throughput screenings as only two samples can be analyzed at the same time 74. Therefore, the Seahorse XF Extracellular Flux Analyzer has been introduced about 10 years ago, offering 24‐ and 96‐well‐based assay platforms. Thereby, the oxygen consumption rate and the extracellular acidification are measured in parallel by fluorescent sensors. However, this brings the limitation that injectable compounds may interfere with the fluorescent sensor. Moreover, in contrast to the Oroboros O2k, only four compounds can be injected during the measurements. Since the sensor‐containing biocartridge has to be loaded with the injectable compounds before starting the actual measurement, it is not possible to change or adapt the compound concentrations during the measurement. Notably, various cell and tissues plates and protocol are meanwhile available, making the analysis of not just adherent cells but also tissues, suspension cells, permeabilized cells, and isolated mitochondria possible 75. Another appealing approach is the usage of genetically encoded (GE) ATP indicators based on a Förster resonance energy transfer (FRET) and equipped with an ATP‐sensing subunit of the bacterial F_O_F_1_‐ATP synthase. Usage of these so‐called ATeams sensors enabled tracking of ATP levels ranging from 7.4 µm to 3.3 mm in different cellular compartments in real time 76, 77.

## Fine‐tuning of mitochondrial activity

### The H^+^ ion

Mitochondrial activity and function are not solely controlled by mitochondrial structure, but also by various fine‐tuning mechanisms, including homeostasis of ions (Fig. 1). In mitochondria, the H^+^ has a unique role. It is well known that the concentration of protons (H^+^) needs to be tightly regulated to preserve essential functions on a single cell level as well as in organisms 78, 79. While the cytosol, the ER, and the nucleus have a neutral pH of 7.0 to 7.4, lysosomes or secretory vesicles maintain an extremely acidic pH for degradation or secretion purposes 80, 81. Strikingly, we can find acidic as well as alkaline areas in mitochondria 82. While the mitochondrial intermembrane space represents a slightly acidic environment with a pH of ~ 6.8, the mitochondrial matrix is the most alkaline compartment within a cell with pH values around 7.6–8.0 82. This difference in the proton concentration is mainly caused by the activity of the respiratory chain, transporting electrons along respiratory complexes. The serial reduction of electrons provides enough energy to shuttle protons via complex I, III, and IV from the matrix into the IMS against their concentration gradient. The accumulation of H^+^ in the intermembrane space is essential for building up a driving force to activate the ATP generating F_1_/F_0_ ATP synthase, while re‐entering the mitochondrial matrix 4, 5, 6. Protons are forced back into the mitochondrial matrix by the pH gradient, a chemical or concentration gradient, and the Δψ_m_, representing a charge or electrical gradient 83, 84.

The Δψ_m_ is not only essential for the generation of ATP 6, but also to regulate transfer of ions like K^+^ 83, 85, 86, Na^+^ 87, 88, Cl^−^ 89, 90, and Ca^2+^ 91 across the mitochondrial membrane. Dysregulation of Ca^2+^ homeostasis and mitochondrial Ca^2+^ overload results in increased permeability of the IMM to protons, decreasing Δψ_m_ as well as the mitochondrial pH gradient 92 and initiating cell death 93.

Since the mitochondrial metabolism is tightly regulated, it is not surprising that dysregulations and sustained changes of the Δψ_m_ lead to severe mitochondrial dysfunctions and have been associated with various disease conditions, including cancer 94, 95 and neurodegeneration 96, 97. For instance, some types of tumors have been associated with elevated Δψ_m_ linked to increased glycolytic rates and resistance to regulated cell death 98. Consistent with these reports, some lung cancer cell lines (A549, H446, SPC), breast cancer MCF7 cells, and glioblastoma MO59K cells exhibited higher Δψ_m_ than the correspondent healthy, noncancerous cell types 94. Moreover, higher Δψ_m_ was linked to increased tumorigenicity and malignancy of cancer stem cells, linked to development and (re)occurrence of malignant tumors, while cells with lower Δψ_m_ were more prone to differentiation. Since reduction of Δψ_m_ by rapamycin decreased tumorigenicity significantly in these cells, targeting Δψ_m_ might be a potential strategy to prevent development of malignant tumors 94.

#### Techniques to measure pH_mito_ and Δψ_m_

Nowadays, a huge variety of indicators and GE sensors is available to determine pH_mito_ and Δψ_m_ and the most prominent candidates will be presented below.

mtAlpHi was described as one of the first encoded pH_mito_ indicators, visualizing and characterizing metabolic changes within mitochondria 99. The calmodulin of the Ca^2+^ indicator camgaroo was substituted by a portion of aequorin comprising only two EF hands, resulting in a Ca^2+^ ‐insensitive probe with an estimated pKa of 8.5, excitation at 498 nm, and emission at 522 nm.

One of the most commonly used GE pH_mito_ sensors is SypHer 92, based on a circular permutated yellow fluorescent proteins (YFP) derived from mutating the cysteine residues of the H_2_O_2_ sensor Hyper 100. SypHer exhibits ratiometric responses at 430 nm and 480 nm upon changing the pH, but is insensitive to H_2_O_2_, Ca^2+^, and PO^3−^. SypHer proved perfectly suitable for the detection of cytosolic as well as mitochondrial pH values due to a pK_a_ of 8.71, a 20‐fold increase upon switching from pH 7 to 10, and a fourfold increase in the range between pH 7 and 8. Furthermore, SypHer has been also used in simultaneous measurements in combination with other sensors performing two‐ or multi‐color imaging 92.

The development of small chemical fluorescent dyes to specifically monitor pH_mito_ has been challenging, but was finally accomplished a few years ago. While dyes like BCECF 101 or SNARF 102 have been used to measure pH in the cytosol, probes like Mito‐pH specifically stain mitochondria. Mito‐pH consists of a pH‐sensitive FITC fluorophore fused to a pH‐insensitive hemicyanine group. This pH‐insensitive part of the probe not only acts as the reference fluorophore for a ratiometric read‐out, but allows for localization in mitochondria, due to their lipophilic cationic nature. The sensor reacts reversibly to changes in pH, thereby exhibiting a double ratiometric read‐out of dual excitation/dual emission and dual excitation between pH 6.1 and 8.4 103. Another approach to monitor pH changes was made by using a chemical system composed of a piperazine‐linked naphthalimide being responsible for the fluorescent off and on signaling, a cationic triphenylphosphonium group for specific mitochondrial targeting, as well as a reactive benzyl chloride subunit for fixation in mitochondria. This probe accumulates within mitochondria due to the effect of the Δψ_m_ on the cationic triphenylphosphonium group. Additionally, the benzyl chloride was thought to undergo nucleophilic substitution with reactive thiols of mitochondrial proteins, ensuring mitochondrial localization even upon acidification or membrane depolarization 104.

While several GE pH sensors have been developed in the last decade, to the best of our knowledge, there is no GE sensor available for measuring Δψ_m_.

Frequently used indicators for semiquantitative analysis of Δψ_m_ are small fluorescent dyes based on a lipophilic cation structure like TMRM, tetramethylrhodamine ethyl ester (TMRE) 105, and 5,5′,6,6′‐tetrachloro‐1,1′,3,3′‐tetraethylbenzimi‐ dazolylcarbocyanine iodide (JC‐1) 106. TMRM and TMRE are single wavelength indicators emitting red light upon Δψ_m_‐dependent accumulation within mitochondria. The excitation and emission wavelength of these monochromatic dyes are quite similar, with excitation at 555 nm and emission at 570 nm for TMRM and excitation at 549 nm and emission at 574 nm for TMRE 105. If used in higher concentrations, TMRM and TMRE might alter the cellular respiration and bind to mitochondrial membranes 105, 107. In contrast, JC‐1 (λ_ex_: 488 nm) is a ratiometric dye, existing as green fluorescent monomer at depolarized membrane potential (λ_em_: 530 nm) and forming red aggregates (λ_em_: 595 nm) at hyperpolarized membrane potential 106, 108. Notably, JC‐1 is very photosensitive and the fluorescence may be changed independently of Δψ_m_ by, for instance, H_2_O_2_ or disturbed equilibrium between monomers and aggregates. All these dyes are differently permeant and require specifically adjusted loading protocols dependent on the respective cell type 108. Moreover, complete depolarization of mitochondria, by for instance FCCP, might be necessary to achieve a baseline, which makes comparison of different Δψ_m_ measurements possible 109.

### The K^+^ ion

Potassium ions (K^+^) are essentially involved in various processes and represent the most abundant intracellular cation. The cytosolic K^+^ concentration is important for the maintenance of the cell's membrane potential, works as cofactor for various enzymes, and regulates cell volume as well as endo‐ and exocytosis. Also, mitochondrial functions rely on an intact mitochondrial K^+^ ($K_{\text{mito}}^{+}$) homeostasis. While cytosolic K^+^ concentration is close to 140 mm in vital cells, the concentration of $K_{\text{mito}}^{+}$ ranges between 20 and 60 mm 110, 111.

Since mitochondrial volume 112, Δψ_m_ 113, mitochondrial metabolism 114, and ROS production 115 are tightly coupled to $K_{\text{mito}}^{+}$_,_ the transport of this ion has to be strictly controlled by K^+^ channels and transporters located in the IMM 116.

Δψ_m_ drives K^+^ influx by diffusion across the membrane, referred to as K^+^ leak, mostly via two‐pore domain K^+^ (K2P) channels, and via ATP‐sensitive K^+^ channels located in the IMM (mitoK_ATP_). Besides, voltage gated K^+^ (Kv) channels and Ca^2+^ activated K^+^ (KCa) channels are located within the IMM 117, 118.

The role of mitoK_ATP_, in particular, has been extensively studied. The generation of high levels of ATP by F_O_F_1_‐ATP synthase causes a decrease in Δψ_m_ and leads to a decreased flux of K^+^ across the mitoK_ATP_ channels into the matrix, probably preventing devastating mitochondrial depolarization. However, under conditions of oxidative stress, increased levels of ROS activate mitoK_ATP_ channels, dissipating Δψ_m_ and counteracting further ROS production. The interplay between K^+^ and H^+^ becomes evident when considering the presence of K^+^/H^+^ transporters (KHX) in the IMM, transporting K^+^ from and H^+^ into the mitochondrial matrix. One of these transporters is the leucine zipper‐EF‐hand‐containing transmembrane protein 1 (LETM1), facilitating K^+^ extrusion from the mitochondrial matrix and, thereby, also modulating Na^+^ and Ca^2+^ homeostasis 119.

High levels of matrix K^+^ assist in modulating the transmembrane H^+^ gradient, altering ATP production, but may also promote the controlled re‐generation of the H^+^ gradient toward the IMM via the ETC 120. Interestingly, the administration of nonselective K_ATP_ channel blockers such as glibenclamide, widely used for the treatment of type 2 diabetes, was found to ameliorate ischemia reperfusion injury in the brain, kidney, intestine, and lungs. The effect was associated with a modulation of the oxidative stress caused by releasing ischemia, after starting reperfusion 121, 122, 123, thus highlighting the importance of proper $K_{\text{mito}}^{+}$ homeostasis.

Besides the interplay of $K_{\text{mito}}^{+}$ with Δψ_m_ and H^+^, $K_{\text{mito}}^{+}$ is fundamentally affected by mitochondrial Ca^2+^ homeostasis. The most prominent example of Ca^2+^ activated K^+^ channels represents the large conductance K_Ca_ (BK_Ca_) channel, found in the plasma membrane of excitable cells such as neurons and skeletal muscle cells. Several types of K_Ca_ channels could be also found in the IMM in various cell types, including the mitochondrial localized large conductance K_Ca_ (mitoBK_Ca_) in LN299 human glioma cells 124. Activation of these channel types is caused by elevated cytosolic Ca^2+^ levels as well as by changes in the Δψ_m_, leading to K^+^ fluctuations across the membrane. While the pore vestibule of mitoBK_Ca_ faces the intermembrane space, the Ca^2+^ sensitive domain is located in the mitochondrial matrix, indicating that activation of mitoBK_Ca_ requires elevation of mitochondrial matrix Ca^2+^ 125, 126, 127. Notably, charybdotoxin, which strongly inhibits BK_Ca_ channels at the plasma membrane, failed to affect mitoBK_Ca_, raising the question whether mitoBK_Ca_ and BK_Ca_ are structurally possibly unrelated to each other 127, 128, 129.

Notably, application of compounds acting as plasma membrane BK_Ca_ channel openers like NS1619 126, accelerating mitochondrial K^+^ uptake twofold, halved the size of a myocardial infarct in guinea pig hearts after reperfusion of ischemic regions. These results suggest the participation of mitoBK_Ca_ channels against necrotic and apoptotic cell injury after ischemic tissue damage, possibly by modulation of the mitochondrial respiratory chain 7, prevention of [Ca^2+^]_mito_ overload, maintenance of Δψ_m_, and/or normalization of ROS levels 126, 130, 131. Moreover, NS1619 as well as CGS7184, another BK_Ca_ channel opener, also exhibited protective effects on neuronal cells 132, 133. Application of these compounds resulted in K^+^ flux from the extracellular space into the mitochondrial matrix in isolated rat brain mitochondria, causing Δψ_m_ depolarization and reduced ROS production 134. In addition, application of the BK_Ca_ channel activator NS11021 was reported to inhibit glutamate‐induced oxidative stress by attenuating ER stress and mitochondrial dysfunction 135. Notably, CGS7184 was shown to directly activate the mitoBK_Ca_ by single‐channel recordings. While this compound boosted oxygen consumption rate in isolated rat brain mitochondria, it exhibited cytotoxic effects by increase of cytoplasmic Ca^2+^ concentration and consequent activation of calpains in intact neuronal HT22 cells 136. These results highlight the therapeutic potential but also the risk for side effects of BK_Ca_ channel modulating compounds.

#### Techniques to measure mitochondrial K^+^

Considering the importance of intact cellular K^+^ homeostasis, scientists have been eager to find ways to investigate cellular and mitochondrial K^+^ levels. $K_{\text{mito}}^{+}$ dynamics are frequently measured via patch‐clamp approaches using isolated mitochondria or mitoblasts 118, 126, 128, 137. A valuable alternative to investigate subcellular and especially $K_{\text{mito}}^{+}$ dynamics within single living cells is provided by fluorescent indicators 138, 139, 140. These indicators represent small chemical dyes, either unspecifically labeling the whole cell or specifically localizing within mitochondria. K^+^ binding to the sensors results in an alteration of their fluorescence depending on the K^+^ concentration 138, 139, 140. Recently, the first GE indicators suitable for intracellular K^+^ measurements have been developed 111, 141. These probes, referred to as GE potassium ion indicators (GEPIIs), in combination with available chemical fluorescent indicators sensitive for K^+^ will in future deepen our understanding of subcellular and particularly mitochondrial K^+^ homeostasis 142.

### The Ca^2+^ ion

While mitochondrial function and activity is heavily dependent on Ca^2+^ homeostasis, mitochondria, in turn, also affect the ion's intricate role as a widespread signaling molecule within the cell. Previously thought to function primarily as a regulator of cytosolic Ca^2+^, it was later determined that ${\text{Ca}}_{\text{mito}}^{2+}$ influx and efflux machinery are geared more toward control of the organelle´s own Ca^2+^ levels 143. Changes to ${\text{Ca}}_{\text{mito}}^{2+}$ concentration are known to have an effect on cellular ATP production 144, 145, respiration 146, ER–mitochondria crosstalk ability (as reviewed by 147), the onset of cellular apoptosis 148, 149, autophagy 150, and many other processes related to cellular health. The mitochondria's function also depends heavily on its ability to send messages to other organelles and receive them from the rest of the cell through Ca^2+^ movement. For example, the mitochondrion is known to preferentially take up Ca^2+^ released by the ER 151, a process that usually involves close contact sites between the two organelles 150. Importantly, such association of the mitochondria with other cellular components is not limited to the ER; rather, other interaction has been documented between mitochondria and the nucleus 152, cytoskeleton 142, and plasma membrane 153, among others. Of the signals that pass to and from these parts of the cell, ${\text{Ca}}_{\text{mito}}^{2+}$ uptake, specifically, has multiple significant consequences pertaining to proper overall function; Ca^2+^ being sequestered into the matrix ultimately has an effect on local and more widespread cellular Ca^2+^ signals. Homeostasis between this organelle´s Ca^2+^ stores and the rest of the cell is crucial, as an overload of intraluminal Ca^2+^ can lead to the initiation of the apoptotic pathway 8.

Mitochondria are able to activate the intrinsic apoptotic pathway following Ca^2+^ overload through the release of multiple proteins from their lumen into the cytosol 154. For example, cytochrome c, an important player in the ETC, is under normal circumstances found attached to the IMM. Following Ca^2+^ overload, mitochondria release this protein into the cytosol, where it stimulates the formation of a caspase‐activating complex otherwise known as the apoptosome. The apoptosome's overall function of activating killer caspases ultimately results in the death of the cell 155. Concerning another important aspect of metabolism and health, cellular respiration, Ca^2+^ is again vital. It is known that the close positioning of mitochondria to large sources of Ca^2+^ (namely, the ER or the plasma membrane) allows for significant accumulation of the ion inside the mitochondrial matrix upon physiological Ca^2+^ release. This increase in matrix Ca^2+^ levels, in turn, affects mitochondrial metabolism through stimulation of mitochondrial effector molecules such as particular dehydrogenases of the Krebs cycle which are functionally dependent on Ca^2+^, ultimately leading to ATP production 156. The ability of mitochondria to accumulate Ca^2+^, and the existence of a ${\text{Ca}}_{\text{mito}}^{2+}$ uniporter, has been reported on since the 1960s 157, 158, but the molecular identity of the MCU was only determined in 2011 159, 160. MCU is a 40 kilodalton (kDa; 35 kDa in its cleaved form) IMM protein with two transmembrane domains, which forms the core of the uniporter complex 159, 160, 161 ${\text{Ca}}_{\text{mito}}^{2+}$ uptake occurs primarily through MCU activity, and this uptake is highly selective but has a low apparent *K*_D_ for Ca^2+^.

These effects are achieved through MCU regulators. Essential MCU regulator (EMRE) was shown to be a core component of the uniporter complex and is essential for MCU mediated‐ MICU 162. In addition, EMRE was shown to regulate MCU activity based on matrix Ca^2+^ levels 163. MICU1 and MICU2 are considered to be MCU gatekeepers, setting a ${\text{Ca}}_{\text{mito}}^{2+}$ uptake threshold, which is higher than that of the MCU 164, 165. Cells with MICU1 knockdown were shown to have elevated basal ${\text{Ca}}_{\text{mito}}^{2+}$ levels 166. MICU2 is considered to be a negative regulator of MCU 165. Thereby, the stoichiometry of MICU1 to MICU2 was shown be an important factor in Ca^2+^ uptake and was also reported to vary across different tissues and organs 167. Besides, also UCP2 and UCP3 were shown to influence MCU‐dependent Ca^2+^ uptake at higher Ca^2+^ concentrations, whereas LETM1 was shown to influence a potentially different uptake mechanism, likely more pronounced when Ca^2+^ levels are lower 168. It was shown that UCP2 normalizes MICU in case of protein methyl transferase 1‐driven methylation of MICU1, resulting in sensitivity loss of MICU1 to Ca^2+^ 169. An important, but as of yet unanswered, aspect of ${\text{Ca}}_{\text{mito}}^{2+}$ uptake is whether there are different uptake pathways operating in relation to low versus high cellular Ca^2+^ concentration sources, as well as what the physiological implications of these potential different uptake pathways may be. Another interesting phenomenon that awaits clarification is the spatial resolution of ${\text{Ca}}_{\text{mito}}^{2+}$ uptake, namely whether it occurs along the full length of the IMM, or whether this activity is restricted to certain membrane regions. Important to consider when discussing uptake of Ca^2+^ into the mitochondrial matrix is the organelle's ability to balance overall charge through extrusion mechanisms. In the case of the mitochondrion, Ca^2+^ influx is mainly kept in check through an exchange with other ions. The major protein playing a role in this process was identified to be Na^+^/Ca^2+^ exchanger (NCLX) 170. Its presence thus necessitates another mechanism to remove the excess Na^+^ that accumulates in the matrix following the exchanger's activity. This is proposed to be achieved by members of the Na^+^/H^+^ exchanger family, consisting of multiple plasma membrane and organellar transporters 171. LETM1 has also been considered as having a role in Ca^2+^ movement, though its exact function in this capacity is as yet unclear. It has been proposed to act as a Ca^2+^/H^+^ antiporter, which implies a function in Ca^2+^ extrusion 172. Other publications indicate that it is instead a K^+^/H^+^ antiporter, thereby influencing Ca^2+^ uptake and/or extrusion via secondary means 119, 173.

#### Techniques to measure mitochondrial Ca^2+^

Mitochondrial Ca^2+^ homeostasis is clearly a complex and wide‐ranging process; therefore, the techniques required are also spread across multiple disciplines. In particular, the use of various sensors to study intracellular Ca^2+^, as well as more specific indicators able to determine the ion's movement to, within and from the mitochondrion itself, provide the quickest insight into the intricate web of Ca^2+^ signals constantly present throughout the cell.

Probes for overall intracellular Ca^2+^ measurement can generally be clustered into chemically engineered fluorophores and GE FPs.

In the recent past, the number of Ca^2+^ sensors specific to mitochondria and other organelles has reached new heights. Sensors used to measure general intracellular Ca^2+^ movement, including the ubiquitously employed cytosolic Ca^2+^ sensor Fura‐2 174, as well as the Calcium green family of indicators, are frequently being supplemented, improved upon or replaced by novel developments in sensor technology that allow for a more direct view of the processes occurring within mitochondria and other organelles. With the huge variety currently available, many criteria must be considered to optimally measure intracellular or mitochondrial Ca^2+^, dependent on the desired experimental read‐out. Choosing the best‐suited Ca^2+^ sensor necessitates consideration of factors such as the probe´s original form and modification required for expression in cells, its affinity for Ca^2+^, and its spectral properties, among others (as thoroughly reviewed by 175).

Briefly, chemical indicators, compounds which change their fluorescence properties following binding to Ca^2+^, are perhaps best suited for observation of the ion's cytoplasmic movement. While they are much simpler to employ than the average GE sensor (no cellular transfection required; simple cell‐loading steps are sufficient), a main disadvantage of chemical sensors for organelle‐specific Ca^2+^ measurement is the lack of controlled localization once loaded into target cells. Use of such probes will therefore not guarantee mitochondria‐exclusive expression and Ca^2+^ monitoring. Chemical fluorophores are thus mainly suitable for drawing indirect conclusions on ${\text{Ca}}_{\text{mito}}^{2+}$ signaling as it pertains to the rest of the cell.

Fura‐2 is a classic example of a ratiometric sensor designed for such purposes. Compared to what was available prior to its characterization, Fura‐2 offered ~ 30× increased fluorescence signals and improved ability to bind Ca^2+^ specifically over other divalent cations, among other advantages 174. This sensor and its variants continue to appear prominently in cellular Ca^2+^ research related to crosstalk between mitochondria and the rest of the cell; for example, investigation into the mechanism whereby the ER and mitochondria interact through mitochondria‐associated ER membranes (MAMs) commonly employs the cytosolic Ca^2+^ sensor Fura‐2 to draw conclusions on Ca^2+^ signaling between the two organelles 176, 177. Fluo‐3 is another fluorescent dye that has been widely used to measure cytoplasmic Ca^2+^ movement, but has its own purported disadvantages. For example, Lee *et al*. 178 showed that in certain cell types this indicator leaks significantly, leading to lowered fluorescence measurements for intracellular Ca^2+^, combined with increased background fluorescence as leaked dye binds to Ca^2+^ present in surrounding medium. Fluo‐3's close relative, Fluo‐4, proves similar in structure and other properties, but exhibits increased fluorescence and range for Ca^2+^ 179. There are also cell‐permeant dyes that target the mitochondria specifically, such as the Rhod‐2 dye of the rhodamine‐based family of indicators. These dyes were first introduced in the late 1980s and include Rhod‐2, X‐rhod‐1, and many variants, all of which exhibit increased fluorescence upon binding Ca^2+^ 180. The AM ester varieties of these indicators are cationic, which causes ion‐potential‐centric uptake of Ca^2+^ into the mitochondria. Due to these properties, the rhodamine‐based AM esters have been employed in the literature as mitochondrial‐Ca^2+^ selective indicators 181, 182. The appeal of Rhod‐2, for example, is easy to see, as just like Fura‐2 and other common cytosolic Ca^2+^ dyes, only cellular incubation prior to experiments is required. However, Rhod‐2 is known to diffuse out of the mitochondria and into the cytosol after a relatively short period of time, making longer experiments unreliable insofar as accurate mitochondrial Ca^2+^ measurement goes. These are but a few examples, and, importantly, each of the outlined tools come with their own advantages and drawbacks. Nevertheless, together, they exemplify the broad range of sensors currently available for cytoplasmic Ca^2+^ measurement.

Among those sensors that are continuously evolving are the new generation of GE fluorescent sensors; compounds comprised of a FP fused to some form of sensing polypeptide. In most cases, the FP is bound to a protein that undergoes a conformational change in response to substrate binding. These sensors are generally considered advantageous over their chemical counterparts due to their substrate specificity, and the fact that their GE nature prevents variance in probe uptake across different cells 183. Touted as being perhaps the most useful advantage of such sensors is the ability to target them to highly specific cellular regions 184, as shown at the turn of the century by Arnaudeau *et al*. (2001) with the use of a cameleon indicator targeted to each of the cytosol, mitochondrial matrix, and ER lumen.

Development of tools for accurate measurement of ${\text{Ca}}_{\text{mito}}^{2+}$ levels specifically has provided new insights into ${\text{Ca}}_{\text{mito}}^{2+}$ fluxes and their regulation.

For example, 4mtD3cpV is a FRET‐based ratiometric Ca^2+^ sensor that is currently widely used. It is excited with blue light (430 nanometers—nm) and exhibits dual emission at 480 and 535 nm. The ratio of the 480 nm emission to the FRET signal (535 nm) provides investigators with insight into and enhanced understanding of basal ${\text{Ca}}_{\text{mito}}^{2+}$ levels as well as its fluctuations 185.

Another FRET‐based ratiometric Ca^2+^ sensor, which can be combined with cytosolic Fura‐2 dye, is 4mtD1GO‐CAM 186. It is a red‐shifted sensor, which allows the researcher to measure Ca^2+^ in the mitochondria while simultaneously employing Fura‐2 to measure cytosolic Ca^2+^. As Fura‐2 has a wide excitation–emission spectrum, combined measurement with 4mtD1GO‐CAM proves extremely useful for investigation into the spatiotemporal fluxes of Ca^2+^. In addition, as both the Fura‐2 dye and mtD1GO‐CAM sensor are ratiometric, accurate observation of basal Ca^2+^ levels is also possible 186.

Ca^2+^ sensors of the GECO series provide a good opportunity to measure Ca^2+^ levels simultaneously in different compartments 187, 188. mtGEM‐GECO1 is a ratiometric sensor with a very low *K*_D_ for Ca^2+^ (340 nm) and has an excitation wavelength of 394 nm and dual emission of 455 and 511 nm 187. Its relative mtCAR‐GECO1 is an intensiometric sensor with excitation and emission spectra of 565 and 620 nm, respectively 188. Excitation and emission spectra of CAR‐GECO1 and GEM‐GECO1 sensors allow measurement of Ca^2+^ levels in two different organelles or mitochondrial compartments simultaneously with almost no spectral overlap.

From this simple examination of a handful of the more prominent cytosolic and mitochondria‐specific sensors in use today, it is clear that investigators have no lack of options when it comes to studying the intricate movements of Ca^2+^ in this organelle and throughout the cell.

## Mitochondria as signaling hubs: Give & Get

### Interplay between mitochondria and nucleus

Although mitochondria are equipped with their own circular deoxyribonucleic acid containing 37 genes (i.e., 13 genes encoding for proteins, such as subunits of the respiration complexes and the ATP synthase, 24 genes encoding for tRNAs), almost all mitochondrial proteins are encoded by the nuclear genome, making constant communication between mitochondria and the nucleus indispensable 10. Consequently, mitochondrial biogenesis strongly depends on the contribution of the nucleus, and properly controlled signaling cascades are required to fine‐tune mitochondrial protein synthesis, counteract mitochondrial dysfunction, and initiate compensatory mechanisms 189. Constant mitochondrial status control by the nucleus helps to prevent mitochondrial malfunction, to counteract damage, and to restore mitochondrial homeostasis via situation‐induced activation of transcriptional response 190. The most prominent example of mitochondrial–nucleus crosstalk is retrograde signaling pathways. In these pathways, the mitochondrial unfolded protein response (mtUPR) initiates a protective transcriptional program upon proteotoxic perturbations, aiming to re‐achieve homeostasis in mitochondrial protein biosynthesis and recover the defective organelle 191, 192.

#### Techniques to measure mitochondrial–nucleus interplay

Dually targeted proteins, localizing to the nucleus as well as to mitochondria, are used as communication indicators for mtUPR (retrograde) signaling. As mitochondrial protein import strongly affects cell viability, the transport of these proteins, visualized by tagging them with a FP, can be used as an indicator of mitochondrial fitness 193, 194, 195. For instance, the mammalian activating transcription factor 5 is imported into mitochondria under physiological conditions, but trapped and consequently translocated to the nucleus to activate transcriptional adaption in case of mitochondrial dysfunction 196. In addition, transcriptional cofactor G protein pathway suppressor 2, another modulator of mtUPR, has also been presented as such an indicator protein, translocating between mitochondria and the nucleus depending on the functionality of mitochondria 197.

Interestingly, cytosolic proteins prone to aggregation are imported into mitochondria for degradation. This translocation of misfolded or aggregated proteins from the cytosol to mitochondria, associated with increased mitochondrial stress levels, can be visualized using split FP techniques 198.

### Interplay between mitochondria and ER

Contact between mitochondria and the ER occurs at very specialized junctions stabilized by MAMs, forming locally restricted signaling hubs to restrict and protect the transfer of lipids and Ca^2+^ between mitochondria and the ER 199. First discovered by electron microscopy in the 1950s 200 but their basic function only described in the 1990s 201, 202, investigation of these structures has been further pushed by the development of cutting‐edge technologies like high‐resolution microscopy over the last 20 years 199. Various proteins stabilizing and modulating mitochondrial–ER interplay, including Ras‐related protein RAB32 203, MFN2 204, or phosphofurin acidic cluster sorting protein 2 205, as well as protein tethering complexes like inositol triphosphate receptor (IP3R), inositol‐requiring enzyme 1 α 206, glucose‐related protein 75 and VDAC, have been identified and characterized 207. Disrupted communication between the ER and mitochondria has been associated with pathological conditions and human diseases 208, such as Alzheimer's disease 209, 210, Charcot‐Marie Tooth 211, Parkinson's disease 212, 213, viral infections 214, cancer 215, diabetes mellitus, and age‐related dysfunction 216.

#### Techniques to measure ER–mitochondrial interplay

Different approaches have been developed to explore the physical and functional sides of ER‐mitochondria tethering. The current state‐of‐the‐art technique to visualize MAMs is electron microscopy, making quantification of the distance between the membranes of the ER and mitochondria, as well as the number of contact sites, possible 217. Coupling electron microscopy with tomography has provided 3D models and information about the structure and plasticity of the contact points between the two organelles 20. While these techniques come with unbeatable resolution, they also bring the disadvantage of fixation, possibly affecting mitochondrial structure. Therefore, large efforts have recently been placed to develop methods that allow the visualization of MAMs in living cells and still provide high spatial resolution. As discussed in our ‘Techniques to analyze structure and shape’ section, advancements in high‐resolution fluorescence microscopy and in the design of organelle‐targeted FP‐tagged proteins or sensors allow to visualize and investigate structural and functional mitochondrial and ER interplay in living cells 216. Moreover, split FP (split‐FP) approaches or so‐called bimolecular fluorescence complementation technology have been used to study mitochondrial–ER interaction 218. Cieri *et al*. (2017) have designed split‐GFP‐based contact site sensors (SPLICS), fusing the GFP_1–10_ moiety to an OMM targeting signal and the GFP_11_ β‐strand to an ER leading peptide and varying the linker length between the targeting signals and the split‐FP to visualize narrow (approximately 8–10 nm) and wide (approximately 40–50 nm) distances between the ER and mitochondria. As soon as the split‐FPs are in close vicinity due to the interactions between proteins fused to each fragment, they from a full fluorescent FP 219. Using split‐FP technology based on GFP, dynamic changes in the structure of MAMs could be visualized and enhanced formation of ER–mitochondrial contact by mitochondrial uncouplers shown 220. Moreover, Harmon *et al*. have developed a split‐FP approach to study mitochondrial–ER interaction based on a YFP Venus by fusing the n‐terminal fragment of Venus128 to a mouse ER–protein and targeting the c‐terminal fragment of Venus to the OMM via the n‐terminal leading peptide of TOMM20. As a result, alterations in the MAM structure in response to ER stress, starvation, and protein level changes could be detected 221.

Recently, Ding *et al*. 222 have developed a novel FP approach using a pair of quenched and nonfluorescent FP‐derived monomers that become a fluorescent heterodimer upon FP association. Alford *et al*. 223 exploited this FP technology and generated probes by fusing one monomer to the C‐terminus of TOMM20 and targeting the other monomer of the dimerization‐dependent FP pair to the ER–membrane via calnexin. Hajnoczky *et al*. exploited the FKBPFRB heterodimerization system by fusing FKBP to an OMM targeting signal and combining FRB with an ER retention signal to enable rapamycin inducible tethering between the two organelles. By using this approach, tethering of the ER and mitochondria results in an increase of already in close apposition located contact sites, rather than creating new organelle contacts 224.

Furthermore, recently developed light‐inducible tethering systems allow the induction of ER–mitochondrial interaction, which facilitates the functional study of ER–mitochondrial contacts 225.

Besides various techniques based on cutting‐edge microscopy, an IP3R–VDAC proximity ligation assay has been developed for the quantification of ER–mitochondria interplay. Proximity ligations assays, as an *in situ* tool, enable the detection of endogenous proteins, protein modifications, and protein interactions with high specificity and sensitivity by using antibodies to detect two unique protein targets 226. Furthermore, Percoll density gradient was used to purify MAMs in order to analyze their composition 227. Functional characterization of MAM regions taking lipid exchange into account has been performed using phosphatidylserine (PS) to phosphatidylethanolamine (PE) conversions or as mitochondrial PS content with regard to lipid transfer 228. Since mitochondria are not governed by classical vesicular trafficking mechanisms, required membrane phospholipids for the mitochondrial membrane biogenesis have to be imported into the organelle. Therefore, mitochondria rely on the efficient supply of lipids from the ER, meaning that biosynthesis of some phospholipids depends on the mitochondria–ER crosstalk. PS, coming from the ER and being directly transferred to mitochondria, is converted in the organelle to PE. Therefore, this reaction can be used for the functional characterization of MAM regions by labeling these phospholipids with radioisotopes 228. Moreover, Ca^2+^ imaging has offered a very nice approach to determine Ca^2+^ exchange between mitochondria and the ER 229.

## Conclusion

As presented in the current review, mitochondria are versatile organelles within eukaryotic cells, which deliver utilizable energy and function as signaling hubs, communicating with various cellular compartments to maintain their own function but also to provide their service. Their structure and shape as well as their activity undergo dynamic changes in order to fulfill their tasks under various conditions in different cellular subdomains. As discussed in this review, investigation into mitochondrial function advanced alongside cutting‐edge technologies, enabling us to gain new insight into subcellular signaling processes. Further development of these methods, as well as completely new technological strategies, will most likely further broaden our understanding in the upcoming years. This might potentially yield the path to unveiling subcellular processes causing still‐incurable diseases and, thereby, help to develop novel treatment strategies. Through that example, it is obvious that constructive teamwork between people with different expertise is increasingly important and is urgently needed to meet the upcoming health‐related challenges in an aging society.

## Conflict of interest

The authors declare no conflict of interest.

## Author contributions

CTM, JR, GZ, SB, HB, ZK, and BG contributed chapters to the manuscript. CTM together with WFG and RM planned the manuscript's structure and content.
